# Standardizing disease-specific quality of life measures across multiple chronic conditions: development and initial evaluation of the QOL Disease Impact Scale (QDIS®)

**DOI:** 10.1186/s12955-016-0483-x

**Published:** 2016-06-02

**Authors:** John E. Ware, Barbara Gandek, Rick Guyer, Nina Deng

**Affiliations:** John Ware Research Group, 10 Wheeler Court, Watertown, MA 02472 USA; Department of Quantitative Health Sciences, University of Massachusetts Medical School, Worcester, MA USA; Measured Progress, Dover, NH USA

**Keywords:** Patient-reported outcomes, Health-related quality of life, Disease-specific measures, Multiple chronic conditions, Item response theory, Norm-based scoring, Validity, Responsiveness

## Abstract

**Background:**

To document the development and evaluation of the Quality of life Disease Impact Scale (QDIS®), a measure that standardizes item content and scoring across chronic conditions and provides a summary, norm-based QOL impact score for each disease.

**Methods:**

A bank of 49 disease impact items was constructed from previously-used descriptions of health impact to represent ten frequently-measured quality of life (QOL) content areas and operational definitions successfully utilized in generic QOL surveys. In contrast to health in general, all items were administered with attribution to a specific disease (osteoarthritis, rheumatoid arthritis, angina, myocardial infarction, congestive heart failure, chronic kidney disease (CKD), diabetes, asthma, or COPD). Responses from 5418 adults were analyzed as five disease groups: arthritis, cardiovascular, CKD, diabetes, and respiratory. Unidimensionality, item parameter and scale-level invariance, reliability, validity and responsiveness to change during 9-month follow-up were evaluated by disease group and for all groups combined using multi-group confirmatory factor analysis (MGCFA), item response theory (IRT) and analysis of variance methods. QDIS was normed in an independent chronically ill US population sample (*N* = 4120).

**Results:**

MGCFA confirmed a 1-factor model, justifying a summary score estimated using equal parameters for each item across disease groups. In support of standardized IRT-based scoring, correlations were very high between disease-specific and standardized IRT item slopes (*r* = 0.88–0.96), thresholds (*r* = 0.93–0.99) and person-level scores (*r* ≥ 0.99). Internal consistency, test-retest and person-level IRT reliability were consistently satisfactory across groups. In support of interpreting QDIS as a disease-specific measure, in comparison with generic measures, QDIS consistently discriminated markedly better across disease severity levels, correlated higher with other disease-specific measures in cross-sectional tests, and was more responsive in comparisons of groups with better, same or worse evaluations of disease-specific outcomes at the 9-month follow-up.

**Conclusions:**

Standardization of content and scoring across diseases was shown to be justified psychometrically and enabled the first summary measure of disease-specific QOL impact normed in the chronically ill population. This disease-specific approach substantially improves discriminant validity and responsiveness over generic measures and provides a basis for better understanding the relative QOL impact of multiple chronic conditions in research and clinical practice.

**Electronic supplementary material:**

The online version of this article (doi:10.1186/s12955-016-0483-x) contains supplementary material, which is available to authorized users.

## Background

Disease-specific measures of quality of life (QOL) have the advantage of frequently being more responsive and clinically useful than generic QOL measures which do not focus on any specific condition [1–3], while generic measures have the advantage of enabling comparisons of QOL burden and treatment benefit across diseases [4, 5]. The advantages of disease-specific measures result in part from achieving specificity by measuring the frequency and severity of specific *symptoms* such as joint pain in arthritis [6] or dyspnea in respiratory disease [7]. Conceptually, however, such symptoms capture QOL only to the extent that they are also quantified in terms of their *impact on life* or its *quality* [8].

To achieve the benefits of both measurement traditions, patient-reported outcome (PRO) surveys have integrated questions about specific symptoms with disease-specific and generic QOL measures [9–13]. Content from generic QOL measures has been incorporated in disease-specific measures to the point that a primary difference between them is whether survey questions make attributions to health in general, a specific component of health (e.g., physical or mental), or a specific disease. With more specific attribution, otherwise generic QOL items have been shown to differ markedly in their validity and interpretation. For example, a Sickness Impact Profile item [14] asking about “*not accomplishing as much as usual at work”* because of “health*”*, modified to make attributions to “physical health” versus “emotional problems”, better discriminated between physical and mental conditions in Medical Outcomes Study (MOS) surveys [15, 16]. However, lack of standardization of QOL content precludes use of disease-specific measures in making comparisons across diseases, and substantial gaps in the representation of QOL content known to be affected by specific diseases remain [17]. The content areas most often represented in widely-used generic QOL surveys are not *all* represented in any disease-specific survey of which we are aware. One noteworthy exception, a survey of QOL impact attributed to headache, comes close to doing so [12].

Disease-specific and generic PROs have a complimentary relationship. For example, generic measures can monitor changes in the physical functioning of patients over time in relation to population norms, regardless of the cause of any change. Disease-specific measures can help determine which conditions accounted most for a patient’s limitations in physical functioning and, therefore, make PROs more useful in outcomes research, predictive studies of health care costs, and everyday clinical practice. In theory, tradeoffs between disease-specific and generic measures are unnecessary. *A new approach to measurement is possible, one that quantifies the impact of each specific condition more broadly in QOL terms but also allows conditions to be compared on a common metric.* This approach uniformly applies disease attributions to the same QOL content for every condition and standardizes scoring metrics across conditions. In other words, one method could achieve the advantages of both disease-specific and generic PROs. To the extent that standardized content and scoring across diseases are justified psychometrically, the new approach could also compliment generic measures in practice settings, by providing clinicians with a sound basis for comparing the relative burden of multiple chronic conditions in disease-specific QOL terms. Underlying this advance in methods are some crucial assumptions.

In pursuit of this approach, the Computerized Adaptive Assessment of Disease Impact (DICAT) project was launched with NIH support [18, 19]. DICAT’s goals were to develop a measure of disease-specific impact that was more comprehensive than existing measures and that could be standardized to enable comparisons across conditions. This paper documents the development of the Quality of life Disease Impact Scale (QDIS®), a standardized disease-specific QOL measure that can be scored in relation to norms for the chronically ill US population. It also presents results from initial tests of psychometric assumptions underlying QDIS construction and its empirical validity in comparison with generic measures.

## Methods

### Item bank development

QDIS was developed on the assumptions that disease-specific attributions can improve the validity of otherwise generic QOL items enough to interpret them as disease-specific measures and that the content of disease-specific measures can be as comprehensive as that of generic measures. Thus, the 49-item QDIS bank included item content selected from words and phrases in widely-used generic and disease-specific surveys, representing ten distinct health-related QOL domains of physical, role and social functioning, mobility, emotional distress and well-being, vitality, sleep, health outlook, cognitive functioning, and quality of life. A major modification to all QDIS items was the change in attribution to a specific disease or condition, as opposed to health in general or no attribution. For example, the QDIS item “In the past 4 weeks, how often did your [CONDITION] limit your physical activities such as walking or climbing stairs?” (where [CONDITION] was a *specific* disease such as angina) was nearly identical to a *generic* physical function item previously validated in the MOS [20] and evaluated, with others, in recent qualitative studies [21, 22]. A second modification was to standardize all QDIS content (item stem and response categories) across diseases.

To benefit as much as possible from prior work, successfully-used descriptions of health impact and operational definitions were adapted for QDIS. One primary source of QDIS content and operational definitions were full-length generic surveys fielded in the Medical Outcomes Study (MOS) [16], which included items from which the 36-item MOS short-form was constructed [15, 23], and their predecessors as documented elsewhere [16, 24–28]. In comparison with the SF-36® Health Survey, QDIS added content capturing disease impact on cognitive function, sleep and quality of life. Another source was item content from seven scales measuring QOL impact attributed to headache pain [29] and subsequent adaptations that changed attributions from headache to other conditions [30–33]. QDIS operational definitions were matched with item content as much as possible to improve validity (e.g., behavioral performance or capacity in measuring physical functioning) and were intentionally varied to measure multiple aspects of disease impact.

Items were standardized to have the same stem structure and 5-choice categorical rating scale and a 4-week recall, the latter to achieve a better time sample of outcomes. The few exceptions were the global QOL items and alternate forms of some items included to enable a cross-walk between alternative methods. In addition to minimizing local dependence and correlated errors due to methods effects, this standardization also facilitates single-item administrations (such as on a PDA) as required for computerized adaptive administrations. Instructions, item stems and response categories were written to be understandable by nearly all US adults. The 49-item bank has a Flesch-Kincaid Grade Level of 7.3 and a Flesch Reading Ease score of 74.7, which equates to a reading level of fairly easy [34].

### Sampling and data collection

Data came from Internet surveys of the US household population with oversampling of the chronically ill, fielded in 2011 in three waves (January-February, July–September, October–December). The DICAT study was approved by the New England Institutional Review Board.

Two independent DICAT samples were recruited. The first (pre-ID) sample, *used in item bank development and evaluation*, included panelists aged 18 and older (*N* = 5418) who previously reported being diagnosed with any of nine conditions within five disease groups: arthritis (osteoarthritis, rheumatoid), chronic kidney disease (CKD), cardiovascular disease (angina, myocardial infarction in past year, congestive heart failure), diabetes, and respiratory disease (asthma, chronic obstructive pulmonary disease). The second sample, *used in QDIS norming*, was a representative sample of the U.S. general population age 18 and older, who had chronic conditions in their naturally-occurring proportions; data from adults who reported one or more of 35 chronic conditions (*N* = 4120 out of 5173 total) were analyzed. Panelists in both samples were recruited from the GfK (formerly Knowledge Networks (KN)) research panel of approximately 50,000 adults, which uses address-based sampling from the U.S. Postal Service’s Delivery Sequence File to represent approximately 97 % of U.S. households. Unlike convenience (“opt-in”) panels, the KN panel is a probability-based sample, as recommended for Internet surveys [35]. It includes cell-phone only households, and panelists who do not have Internet access are provided with a computer and Internet connection [36]. Pre-ID conditions for panelists in the first sample were confirmed at the start of the DICAT survey, and pre-ID panelists were sampled to achieve at least 1000 respondents within three disease groups, with smaller targets for less prevalent diagnoses (CKD, cardiovascular).

Panelists were recruited via a routine invitation from KN. E-mail and IVR telephone reminders were sent to non-responders. In the pre-ID sample, 9160 panelists received invitations, of whom 6828 opened the informed consent screen (consent screening rate of 74.5 %), 5585 consented, and 5418 completed surveys (survey completion rate of 97.0 %) [37]. Corresponding figures for the general population sample were 10,128 invites, 6433 (63.5 %) opened the informed consent screen, 5332 consented, and 5173 completed surveys (97.0 %). Responsiveness analyses used Wave 3 data from the pre-ID sample; 2816 Wave 1 pre-ID panelists were invited to complete a Wave 3 survey, of whom 2447 opened the informed consent screen (86.9 %), 2442 consented, and 2384 completed surveys (97.6 %). By design some Wave 3 respondents did not complete a generic SF-8™ Health Survey; a total of *N* = 1889 panelists had QDIS and SF-8 data in both Waves 1 and 3.

Random assignment to survey protocols enabled administrations of different survey modules while controlling respondent burden (median total time ≤ 25 min). For pre-ID and general population samples, the presentation of each QDIS and other items was identical and modules were administered in the following order: generic QOL measures, 35-item chronic condition checklist, 11–49 QDIS items for one pre-ID condition (pre-ID sample only) and 1–11 QDIS items for other conditions endorsed, and legacy disease-specific measures (pre-ID sample only). A total of 4028 pre-ID Wave 1 and Wave 2 panelists completed 49 QDIS items in the same order for their pre-ID condition, while 1390 randomly chosen pre-ID Wave 2 panelists completed only 11 QDIS items for their pre-ID condition. All QDIS administrations began with the same item (everyday activities or quality of life, see Table 2). Other measures used in cross-sectional validation and responsiveness comparisons included the generic SF-8 physical (PCS) and mental (MCS) component scores, which have been shown to correlate highly with the SF-36 Health Survey PCS and MCS [23], along with widely-used disease-specific measures [6, 7, 11, 38–41] matched to each pre-ID condition (see Table 6). Internet data collection allowed data quality to be monitored in real time; accordingly, completeness of survey responses was not an issue. QDIS items had missing data rates of 0.6 to 1.4 %.

**Table 1:** Characteristics of pre-identified and general population chronically ill samples

	Pre-Identified Disease Group					Pre-Identified Combined (*N* = 5418)	Gen. Pop. Chronically Ill (*N* = 4120)
	Arthritis (*N* = 1574)	CKD (*N* = 299)	Cardiovascular (*N* = 639)	Diabetes (*N* = 1326)	Respiratory (*N* = 1580)		
Age							
Mean (SD)	62.1 (11.2)	63.5 (12.8)	65.7 (10.7)	60.3 (11.9)	53.0 (16.1)	59.5 (13.7)	50.9 (16.0)
Median	63	64	66	61	55	61	52
Range	20–93	18–92	18–97	18–92	18–93	18–97	18–94
% Male	35.5	51.8	56.6	52.0	35.2	42.8	47.0
Race/Ethnicity							
White non-Hispanic	82.5	81.6	84.5	77.6	77.0	79.9	77.3
Black non-Hispanic	6.9	8.0	5.3	11.1	7.3	7.9	8.9
Hispanic	5.3	5.0	2.7	6.2	7.6	5.9	8.4
Other non-Hispanic	5.3	5.4	7.5	5.1	8.1	6.3	5.4
Education							
< High school graduate	2.6	4.7	5.6	2.6	3.4	3.3	7.3
High school graduate	20.5	11.7	16.3	19.1	17.8	18.4	26.5
Some college	34.1	41.8	41.9	39.7	38.2	38.0	31.6
College graduate	42.8	41.8	36.2	38.6	40.6	40.3	34.6
Income							
< $20,000	11.6	15.1	14.9	10.6	13.7	12.5	13.8
$20,000–39,999	21.6	24.4	26.8	21.9	18.7	21.6	21.4
$40,000–99,999	47.4	44.8	47.4	52.0	48.0	48.6	41.8
$100,000+	19.4	15.7	10.9	15.5	19.6	17.3	23.0
Employment Status							
Employed	35.9	23.7	20.0	43.4	47.0	38.4	52.4
Unemployed	4.0	1.7	2.8	4.8	5.1	4.3	7.0
Retired due to age	38.0	40.5	42.4	33.0	21.1	32.5	19.3
Disabled^a^	12.8	24.4	26.5	10.2	12.3	14.3	7.7
Other^b^	8.7	8.7	7.4	7.7	13.9	9.8	13.1
Missing	0.6	1.0	0.9	0.8	0.5	0.7	0.5

Arthritis includes osteoarthritis (*N* = 1066) and rheumatoid arthritis (*N* = 508). Cardiovascular includes angina (*N* = 214), recent myocardial infarction (*N* = 98), and congestive heart failure (*N* = 327). Respiratory includes asthma (*N* = 1175) and COPD (*N* = 405)

*Abbreviations: CKD* chronic kidney disease

^a^Retired due to disability

^b^Homemaker, student, other

### Analyses

Evaluation of the 49-item bank included item descriptive statistics, confirmatory factor analyses (CFA) to test the psychometric assumptions of unidimensionality underlying a one-factor model, and multi-group CFA (MGCFA) to test the equality of factor loadings and thresholds underlying the adoption of standardized scoring across diseases. As recommended [42], psychometric assumptions were first tested separately in each of the five disease groups and then for all groups combined. Because satisfying “psychometric” standards of scale construction does not guarantee improved validity for purposes of measuring disease-specific outcomes, QDIS and generic measures were compared in cross-sectional and longitudinal tests to evaluate discriminant validity and responsiveness.

### Unidimensionality and item local dependence

Based on prior research [13, 29, 43–45], a 1-factor model was hypothesized and evaluated for the 49-item QDIS item bank using CFA with a robust weighted least squares (WLSMV) estimator and Mplus [46]. To test whether items were sufficiently unidimensional, the percentage of variance accounted for by one factor was estimated, and model fit was evaluated using the following criteria: comparative fit index (CFI: >0.95 indicating good fit) and root mean square error of approximation (RMSEA: <0.06 recommended, although values are often higher with large samples [47]). Measurement invariance was evaluated by testing the fit of nested MGCFA models without (test of equal forms) and with (test of measurement invariance) the constraint of equal factor loadings and thresholds across groups, using the DIFFTEST option in Mplus [46] and the percentage change in CFI [48]. Plots of disease-specific and standardized (across groups) factor loadings and thresholds also were inspected. Noteworthy residuals (*r* > 0.20, absolute value) were flagged to identify any notable shared item variance not accounted for by the 1-factor model which might indicate item local dependence, as in previous analyses [49].

### Differential item functioning

Ordinal logistic regression (OLR) methods [50] were employed to test for uniform and non-uniform differential item functioning (DIF) for groups differing in age (<65, 65+) and gender, using the R software package lordif [51]. Meaningful DIF was identified by a change in Nagelkerke's R^2^ (for uniform plus non-uniform DIF) >0.03 [52] or a proportionate change >5 % in the beta coefficients (β_1_) for the trait measured (disease impact) in OLR models with and without the group variable [53].

### Item Response Theory (IRT) modeling

IRT parameters were estimated independently for each group (disease-specific parameters) and the combined sample (standardized parameters) using a Generalized Partial Credit Model (GPCM) [54] as in previous studies [17, 55–57] and Xcalibre™ 4 software [58]. Goodness-of-fit was evaluated using chi-square item fit statistics [59] acknowledging that IRT models for polytomous items may misfit even when violations are very small [60]. To evaluate item-level invariance across independent groups, scatterplots for item slopes and thresholds were compared for 10 unique pairs of five disease groups using ResidPlots [61]. In addition, because insufficient item-level invariance may be without practical consequences [60] or may be offset by opposite effects [17], scale-level scores estimated using disease-specific and standardized item parameters were evaluated in each group using product–moment correlations and graphical scatterplots as in a parallel study [17].

### Item bank reduction and short form development

A 25-item bank was developed from the 49-item bank for use in CAT administrations when the cost of item translations and of recording items for oral administrations are important considerations. Selection of items for the shorter bank and a 7-item short form (QDIS-7) was based on the following criteria (ordered in terms of priority): (a) comprehensive representation of content areas; (b) steep item slope; (c) incremental validity in predicting total bank theta score; (d) utilization in real-data CAT simulations of the 49-item bank [62]; (e) item information functions indicative of reliability over a wide range of theta values, and (f) item fit statistics. Content representation without redundancy was a priority. When multiple items represented the same content area and achieved nearly equal discrimination (slope) and incremental validity in predicting theta, higher rates of CAT utilization and higher item information over a wider range determined choices between them. As illustrated in Results, lack of fit in terms of traditional fit statistics was ignored in favor of item usefulness (discrimination, CAT utilization).

The 7-item static (QDIS-7) and adaptive 6-item CAT (QDIS-CAT-6) short forms were both scored using standardized IRT parameters and norm-based algorithms (see below). Because these forms were very highly correlated at baseline (*r* = 0.97–0.98 across five disease groups) and yielded virtually identical results in cross-sectional tests of discriminant validity, results are reported for QDIS-7. Baseline results for QDIS-CAT-6 are reported in Additional file 1: Table S1 and Additional file 2: Table S2.

### Reliability

IRT estimates of the precision of scores at the *person-level* were compared across severity levels (none/mild, moderate, severe/very severe). At the *group-level*, internal consistency reliability (Cronbach’s alpha (1951) [63]) and test-retest reliability were estimated. Test-retest reliability was estimated using intraclass correlation coefficients (ICC) in a sample of Wave 2 panelists including approximately equal numbers from each disease group and severity level, who completed a second QDIS survey within two weeks.

### Validity

To test the hypothesis that changing item attributions from health in general to a specific disease improves the validity of QDIS as a disease-specific measure, one-way analyses of variance (ANOVA) compared scores for QDIS and generic SF-8 measures across identical groups reporting different severity levels within each disease. For the combined pre-ID groups, sample sizes were sufficient for ANOVAs across all five severity levels. The ratio of F-ratios [64] was used to calculate relative validity (RV) and differences in RV were tested for significance as documented elsewhere [65]. We hypothesized that QDIS-7 would discriminate across disease severity groups better than generic measures. In addition, product–moment correlations were estimated to test the hypothesis that correlations with legacy disease-specific QOL measures would be higher for QDIS than for generic SF-8 measures.

### Responsiveness

To extend initial tests to include responsiveness, analysis of variance (ANOVA) compared self-evaluated transition (SET) groups in terms of change scores (9-month follow-up minus baseline) for QDIS and generic SF-8 measures. SET groups were formed from responses (much better, somewhat better, about the same, somewhat worse, much worse) to the question “Compared to 9 months ago, how much better or worse is your < DISEASE > now?”, where DISEASE was the pre-ID condition. Because simulation studies showed that more reliable scores were most useful for the more severely chronically ill, Wave 3 QDIS scores were estimated from a single-item (QL1) supplemented by a 1–9 item CAT only for those with severe QOL impact (QL1 score of “a lot” or “extremely”) [66]. QDIS scores estimated using this method had a high correlation (*r* = 0.90) with the static QDIS-7 and nearly identical means (50.03 vs 49.97, respectively, for QDIS-7 and adaptive CAT) in evaluations of Wave 1 data. ANOVA F-statistics for Wave 3 SET groups were compared using RV estimates as above [65]. Because groups were defined based on evaluations of change in the pre-ID condition, we hypothesized that QDIS-7 would be more responsive than generic SF-8 measures. Results are reported here for all conditions combined.

### Norm-based T-score transformation

IRT-calibrated QDIS scores were transformed to have a mean of 50 and standard deviation (SD) of 10 using a linear T-score transformation of scores for all US general population sample respondents reporting any chronic condition (*N* = 4120). A higher score indicates greater disease impact (worse health). Independent observations, one per person, were weighted using KN-derived sampling weights to adjust the sample to the demographic distribution of the December 2010 Current Population Survey [67]. Norm-based estimates of central tendency, variability and percentile ranks for QDIS-7 were examined. For the combined pre-ID groups, sample sizes were sufficient to estimate norms for each of five severity levels.

## Results

### Sample characteristics

Characteristics of the pre-identified disease samples and chronically ill norming sample are summarized in Table 1. Except for the respiratory group, pre-ID samples tended to be older than the normative sample. The latter sample was also more likely than the pre-ID samples to be employed, but was less highly educated on average. Other characteristics were similar. The characteristics of the subset of pre-ID respondents followed longitudinally did not materially differ from those documented in Table 1.

### Item descriptive statistics

Table 2 summarizes item wording, content classifications, sample sizes, item-level descriptive statistics and other results (discussed below) for the combined pre-ID sample. N for most items differed only slightly (*N* = 3995–4044); exceptions included the global quality of life (QL1) and 10 other items randomly administered more often (*N* = 5372–5399). Underlying consistently low means were large proportions (39–82 %, median = 60 %) endorsing the lowest impact response category.

**Table 2:** Item content and statistics, 49-item disease-specific QOL impact bank

Order^a^	Area^b^	Abbreviated Content^c^	N	Mean (SD)	r^d^	a^e^	b Range^e^	Fit^f^	Imax at Θ^g^	CAT^h^
1^**^	QL	Everyday activity, QOL^i^	5399	1.80 (1.04)	.956	3.55	0.18–2.11	191.9	6.26 (1.20)	100
44	QL	Activity enjoyment life^i^	4044	1.79 (1.03)	.946	2.63	0.20–2.28	151.0	4.20 (1.10)	0
2^**^	HO	Worry health future	5372	2.11 (1.13)	.844	1.49	−0.10–1.86	371.9	1.90 (1.35)	42
36^*^	HO	Concern worry	4022	1.90 (1.08)	.868	1.84	0.13–1.97	284.2	2.65 (1.55)	28
10^**^	PF	Usual physical activities	4019	1.96 (1.12)	.920	3.10	0.00–1.93	158.2	4.76 (1.45)	55
3^*^	PF	Physical activity walking	5386	1.95 (1.20)	.876	2.20	0.25–1.64	148.5	4.11 (1.15)	0
47^*^	MB	Stay inside house	4008	1.57 (1.04)	.894	2.41	0.87–1.94	132.5	5.60 (1.20)	0
27^**^	RL	Work daily activities	4015	1.87 (1.09)	.964	5.30	0.08–1.89	114.4	9.67 (1.40)	64
5^*^	RL	Everyday activities	5381	1.77 (1.06)	.957	4.67	0.26–1.96	101.6	8.64 (1.50)	41
15^*^	RL	Get done work home	4026	1.91 (1.14)	.940	3.88	0.11–1.83	123.6	6.97 (1.50)	14
17^*^	RL	Cancel work activity	4015	1.42 (0.85)	.921	3.47	0.88–2.17	71.9	7.46 (1.95)	2
23^*^	RL	Limited usual activities	4006	1.77 (1.04)	.948	4.50	0.20–1.97	89.2	8.70 (1.65)	14
26^*^	RL	Accomplished less	5395	1.97 (1.16)	.937	3.88	0.07–1.78	85.9	6.94 (1.40)	28
30^*^	RL	Leisure activities	4017	1.35 (0.75)	.873	2.47	1.05–2.49	152.9	4.35 (2.15)	<1
33^*^	RL	Productive work other	4012	1.54 (1.00)	.945	4.12	0.72–1.96	44.9	9.81 (1.00)	26
49^*^	RL	Daily work in and out	3995	1.84 (1.09)	.954	4.52	0.14–1.88	101.5	8.73 (1.60)	34
8	RL	Daily tasks help	4020	1.72 (1.06)	.856	1.93	0.55–1.94	183.1	3.37 (1.35)	0
13	RL	Usual daily activities^j^	4020	1.73 (1.01)	.941	4.14	0.25–2.14	107.0	7.12 (1.30)	0
19	RL	Stop work other activity	4012	1.75 (1.02)	.911	3.09	0.27–2.05	89.1	5.38 (1.75)	0
35	RL	Simple tasks hard	4006	1.81 (1.08)	.923	3.58	0.22–1.93	104.6	6.48 (1.60)	0
38	RL	Keep from traveling	4017	1.46 (0.97)	.916	2.42	1.11–1.95	177.0	6.32 (1.30)	0
41	RL	Restrict recreational	4014	1.48 (0.90)	.897	2.64	0.87–2.17	122.5	5.04 (1.20)	0
42	RL	Cut down amount time	4011	1.68 (1.04)	.942	3.97	0.43–1.97	72.7	7.73 (1.70)	<1
6^**^	SA	Social family friends	5383	1.56 (0.96)	.950	4.56	0.57–2.04	107.1	9.69 (1.75)	30
37^*^	SA	Enjoying social activities	4007	1.60 (0.98)	.945	4.34	0.52–2.08	93.3	8.29 (1.65)	19
45^*^	SA	Hard to get along with	4006	1.52 (0.87)	.853	2.05	0.75–2.43	208.3	3.26 (1.65)	<1
9	SA	Enjoyment family friends	4020	1.63 (0.98)	.915	3.05	0.50–2.19	93.0	5.16 (0.90)	0
14	SA	Avoid social activities	5399	1.55 (0.97)	.938	3.59	0.68–2.12	67.9	7.52 (1.00)	0
21	SA	Uncomfortable people	4015	1.43 (0.85)	.890	2.57	0.93–2.09	135.7	5.54 (1.85)	0
32	SA	Travel take trip	4012	1.52 (1.02)	.915	2.48	0.98–1.82	75.8	6.53 (1.30)	0
40	SA	Family social leisure^k^	4015	1.40 (0.80)	.920	3.33	0.92–2.60	81.8	6.73 (1.10)	1
4^**^	FT	Worn out, tired	5384	1.94 (1.13)	.923	3.41	0.06–1.81	96.5	6.17 (1.45)	19
28^*^	FT	Bed most of day	4018	1.30 (0.73)	.878	2.37	1.34–2.42	124.8	5.23 (1.60)	<1
18	FT	Need lie down rest	4012	1.92 (1.12)	.884	2.44	0.16–1.92	124.9	3.71 (0.55)	0
46	FT	Too tired to work^j^	4007	1.70 (0.98)	.932	4.01	0.26–2.22	107.4	6.70 (1.35)	<1
20^*^	SL	Good night sleep	5387	1.94 (1.14)	.810	1.52	0.31–1.86	294.0	2.27 (1.15)	26
11^**^	EM	Frustrated fed up	4011	2.03 (1.21)	.871	1.99	0.14–1.74	209.3	3.12 (0.70)	36
7^*^	EM	Bothered emotionally	5378	1.67 (1.00)	.906	2.04	0.53–2.00	117.4	3.71 (1.60)	0
16^*^	EM	Interest enjoyment	4018	1.81 (1.07)	.940	4.26	0.20–2.01	110.6	7.19 (0.60)	17
22	EM	Losing control life	4006	1.56 (0.97)	.898	2.35	0.78–1.99	89.0	4.40 (1.70)	0
24	EM	Tense feel anxious	4007	1.67 (0.99)	.895	2.37	0.46–2.07	107.1	4.07 (1.70)	0
29	EM	Fed up frustrated^j^	4032	1.68 (0.99)	.895	2.39	0.39–2.14	167.8	4.29 (1.45)	0
31	EM	Letting others down	4019	1.58 (1.03)	.914	2.86	0.78–1.89	64.1	6.42 (1.05)	0
34	EM	Angry act irritable	4001	1.63 (0.98)	.885	2.09	0.61–2.03	111.8	3.71 (1.65)	0
39	EM	Feel desperate	4009	1.38 (0.82)	.904	2.43	1.08–2.25	114.5	5.25 (1.60)	0
43	EM	Depressed sad	5388	1.62 (0.99)	.911	2.04	0.71–1.98	134.1	3.84 (1.50)	0
25^*^	CG	Difficult to focus	4009	1.53 (0.89)	.921	3.38	0.60–2.20	91.7	6.63 (1.95)	<1
12	CG	Focus on work	4010	1.74 (1.02)	.927	3.68	0.29–2.09	51.6	6.24 (1.70)	0
48	CG	Ability to concentrate^j^	4004	1.51 (0.86)	.923	3.66	0.60–2.36	69.3	6.48 (2.05)	3

^a^Original order of administration in 49-item bank. ^*^In 25-item bank. ^**^In 25-item bank and 7-item short-form

^b^Content area: *QL* = quality of life, *HO* = health outlook, *PF* = physical functioning, *MB* = mobility, *RL* = role functioning, *SA* = social activity, *FT* = fatigue, *SL* = sleep, *EM* = emotional, *CG* = cognitive

^c^All items used 5-choice (Never-Very often) categorical rating scale except: ^i^5-choice (Not at all-Extremely) scale; ^j^Cross-calibration item used 5-choice (None-All of the time) and (Never-Very often) scales in different waves; ^k^Item used 5-choice (Never-Always) scale

^d^Correlation with factor in 1-factor confirmatory factor analysis, all diseases combined (*N* = 3152)

^e^Slope (a) and range of thresholds (b) in IRT model, all diseases combined

^f^S-*X* ^2^ fit statistic, values above 118.75 are significant at a .05 level

^g^Imax at Θ is maximum of the item information function (first number) at a particular theta (number in parentheses)

^h^Percentage of times item utilized in 6-item real-data CAT simulation using 49-item bank. Item QL1 was the start item for the CAT

### Unidimensionality and item local dependence

In support of a generalizable 1-factor model, one dominant factor explaining 80–86 % (median = 84 %) of the total variance was observed across the five disease groups. Item factor loadings were consistently very high in the combined sample (0.81–0.96; median = 0.92) and each disease group (Table 3). The CFI was satisfactory in all five disease groups (0.979–0.991) and combined sample (0.982), and the RMSEA was acceptable (0.058–0.077 across diseases; 0.074 in combined sample). Fit of the equal form (*χ*^2^(5635) = 20,023.4, CFI = 0.987, RMSEA = 0.064 (90 % CI 0.063,0.065)) and measurement invariance (*χ*^2^(6411) = 20,927.7, CFI = 0.987, RMSEA = 0.060 (0.059,0.061)) multi-group CFA models was satisfactory. While the Mplus DIFFTEST for the two models was significant (*χ*^2^_diff_ (776) = 2583.5, *p* < 0.0001), it is known to be sensitive to sample size [48] and the CFI did not differ (to the nearest thousandth) between the two models. Further, examination of modification indices did not support respecification of the model. Accordingly, the 1-factor solution was accepted across disease groups. Almost no evidence of item local dependence was observed among 1176 residual correlations in each of the five groups and combined sample (total of 7056 residuals); only one residual correlation (HO2/HO36, *r* = 0.202) in one disease (CKD) exceeded 0.20. Thus, no items were eliminated from the 49-item bank.

**Table 3:** Summary of item-level CFA and IRT evaluations by disease group and all diseases combined and disease-specific versus standardized inter-scale correlations^a^

Disease	Confirmatory Factor Analysis						IRT parameters		Item r with theta score^d^	Inter-scale correlations^e^
	Χ^2^	df	CFI	RMSEA (90 % CI)	Factor Loadings^b^	Residuals^c^	Slopes	Thresholds		
Arthritis	7037	1127	0.979	0.077	0.91	0.026	2.58	0.99	0.76	0.9996
				(0.076–0.079)	0.74–0.94	0.00–0.143	1.18–4.02	−0.83–2.72	0.59–0.86	
CKD	1865	1127	0.991	0.058	0.93	0.026	3.55	1.36	0.76	0.9983
				(0.053–0.062)	0.76–0.97	0.00–0.202	1.29–5.94	−0.24–2.38	0.62–0.83	
Cardiovascular	3658	1127	0.987	0.069	0.92	0.022	3.13	1.18	0.75	0.9998
				(0.067–0.072)	0.81–0.96	0.00–0.141	1.62–5.51	−0.20–2.71	0.59–0.86	
Diabetes	3991	1127	0.985	0.061	0.91	0.027	2.98	1.85	0.70	0.9992
				(0.059–0.063)	0.81–0.97	0.00–0.186	1.36–6.05	−0.29–2.99	0.51–0.81	
Respiratory	4847	1127	0.990	0.060	0.92	0.021	3.13	1.50	0.75	0.9999
				(0.058–0.061)	0.84–0.97	0.00–0.124	1.64–5.87	0.10–2.67	0.58–0.86	
Combined	20,669	1127	0.982	0.074	0.92	0.023	3.05	1.34	0.74	-
				(0.073,0.075)	0.81–0.96	0.00–0.158	1.49–5.30	−0.10–2.60	0.58–0.85	

*Abbreviations: Χ* ^*2*^ chi-square test of model fit, *df* degrees of freedom, *CFI* comparative fit index, *RMSEA* root mean square error of approximation, *CKD* chronic kidney disease

^a^All entries are median on first line and minimum-maximum on second line unless noted

^b^Loadings from the completely standardized CFA solution for each disease (rows 1–5) or all diseases combined (row 6)

^c^Absolute value of residual correlations after controlling for single factor for each disease (rows 1–5) or all diseases combined (row 6)

^d^Correlation between person-level item scores and total bank theta scores for each disease (rows 1–5) or all diseases combined (row 6)

^e^Correlation between person-level total bank theta scores estimated using disease-specific and standardized item parameters

### Differential item functioning

There was no noteworthy DIF by age or gender. The median change in Nagelkerke’s R^2^ was ≤0.001 across items for both age and gender and the highest ΔR^2^ was 0.006, well below the threshold ΔR^2^ of 0.03. Similarly, the median percentage increase in β_1_ was <0.3 % for both age and gender and the maximum increase was 3.3 %, below the threshold of 5 %.

### IRT and other item-level properties

Table 2 summarizes IRT and other item-level properties for all 49 items, including IRT slopes and factor loadings, which are directly related [42]. It identifies (**) seven items chosen for QDIS-7 with the following abbreviated item content (content area designation in parentheses): everyday activities or quality of life (QL), worry about health in future (HO), limited in usual physical activities (PF), difficulty in work or daily activities (RL), limited in usual social activities with family, friends or others (SA), worn out or tired (FT), and frustrated or fed up (EM). Table 2 also identifies 25 items recommended for use in CAT administrations.

Item thresholds were most often positive due to skewness and were consistently ordinal for each item (data not reported). Item slopes and CAT utilization agreed considerably, while item fit and CAT utilization tended to disagree; for example, only one of 10 best fitting items was in the top 10 in CAT utilization and five of the latter were among the 10 worst fitting items. The first item in Table 2, QL1, was selected as the first item for QDIS-7 and CAT administrations based on face validity (“everyday activity” and “quality of life” content), a wide range of thresholds, one of the least skewed response distributions, and a very high CFA loading, even though it had a lower slope than many items. Items chosen for QDIS-7 generally included the best item from each of the seven represented content areas in regressions testing incremental validity in relation to the total bank theta score. One exception was the QDIS-7 role functioning item, which was the second best predictor from that content area but was selected because it had much higher CAT utilization. If based entirely on item-level fit (“Fit” in Table 2), most QDIS-7 items would not have been chosen for that static short-form. It is noteworthy that “misfitting” (by traditional standards) items often had very high factor loadings, were among the best predictors of the theta score, and were among the items most often utilized by CAT.

Medians and ranges of disease-specific and standardized (combined) QDIS item slopes were very similar and highly correlated (*r* = 0.88–0.96) as were threshold parameters (*r* = 0.93–0.99) across disease groups (Table 3, Additional file 3: Figure S1 and Additional file 4: Figure S2). This pattern was confirmed across all 10 pairs of slopes (*r* = 0.79–0.89) and thresholds (*r* = 0.90–0.98) estimated independently for the five disease groups (Additional file 5: Table S3). Median item-theta score correlations were very high (*r* = 0.70–0.76 across disease groups), and their ranges also were similar across groups (Table 3). As shown in the plots in Additional file 3: Figure S1 and Additional file 4: Figure S2, estimates of thresholds were more robust than slopes across standardized and disease-specific estimates but both demonstrated substantial linearity.

### Person-level scale scores

Scale-level (theta) scores estimated using standardized and disease-specific item parameters were very highly correlated (*r* > 0.99) (Table 3) and showed near perfect linearity in plots for each of the five groups (Additional file 6: Figure S3). These results support the notion that any differences between disease-specific and standardized item parameters affecting scale-level scores in one direction were offset by differences in other items in the opposite direction.

### Reliability

Internal consistency reliability was consistently high for QDIS-7 scores (alpha = 0.91–0.94) across diseases (Table 4). The percentages of person-level IRT reliability estimates exceeding 0.90 were very high (72–100 %, median = 93 %) for the moderate and severe groups, for whom precision is most important clinically, but lower (34–60 %) for none/mild groups. For the 376 panelists completing QDIS-7 retest surveys within 5–14 days (median = 8 days) and reporting their health was the same, test-retest reliability was satisfactory (ICC = 0.83–0.91 across groups).

**Table 4:** Comparison of QDIS-7 means and reliability estimates, five disease groups

Disease	Mean (SD)^a^	% with IRT Reliability ≥0.90 by Severity Level^b^				Alpha^c^	Test-retest reliability^d^
		Mild	Moderate	Severe	Total		
Arthritis	54.24 (8.72)	(*N* = 718)	(*N* = 587)	(*N* = 231)	(*N* = 1536)	.94	.88
		60.5	93.5	95.7	78.4		
CKD	48.97 (9.17)	(*N* = 200)	(*N* = 61)	(*N* = 36)	(*N* = 297)	.94	.91
		34.5	82.0	94.4	51.5		
Cardiovascular	51.65 (9.87)	(*N* = 483)	(*N* = 112)	(*N* = 36)	(*N* = 631)	.94	.90
		55.5	95.5	91.7	64.7		
Diabetes	47.60 (7.75)	(*N* = 921)	(*N* = 329)	(*N* = 63)	(*N* = 1313)	.91	.83
		38.6	72.0	100.0	49.9		
Respiratory	48.45 (9.45)	(*N* = 1152)	(*N* = 306)	(*N* = 112)	(*N* = 1570)	.94	.88
		36.4	87.3	93.8	50.4		

*Abbreviations: CKD* chronic kidney disease

^a^Analysis of variance indicated that QDIS-7 group means differed across disease groups (F_(4,5413)_ = 152.1, *p* < 0.0001). QDIS-7 scores have mean = 50, SD = 10 in chronically-ill US general population; higher scores equal worse health

^b^Percent with IRT estimated reliability ≥0.90. Severity defined as Mild (None, Mild), Moderate, or Severe (Severe, Very Severe) in response to item *How would you rate the severity of your < condition > in the past 4 weeks?*

^c^Internal consistency reliability for arthritis (*N* = 1113), CKD (*N* = 261), cardiovascular (*N* = 578), diabetes (*N* = 857) and respiratory groups (*N* = 1156)

^d^Intraclass correlation coefficient (ICC_(3,1)_) for arthritis (*N* = 109), CKD (*N* = 37), cardiovascular (*N* = 63), diabetes (*N* = 75) and respiratory groups (*N* = 92)

### Validity

In cross-sectional tests of validity in discriminating across severity levels, QDIS means increased progressively with greater disease severity in all five diseases (Table 5). In support of the hypothesis that disease-specific QDIS measures were more valid than generic measures, a significantly (*p* < 0.05) higher F-ratio for mean differences across severity levels was observed for QDIS-7 (RV = 1.0) in comparison with SF-8 physical and mental measures (RV = 0.05–0.65, median = 0.18) in every disease group. Increases in both mean severity level separations and reductions in within group variances for QDIS-7 contributed to these results. The orders of magnitude of F-statistics for QDIS were markedly larger (1.5 to 20 times, median = 5.6 times) than those observed for generic measures in the same comparisons. In tests of convergent validity, correlations between QDIS-7 scores and corresponding legacy QOL measures for the same disease were very high (*r* = 0.71–0.83) (Table 6). A similar pattern of moderate to high correlations (*r* = 0.54–0.74, median = 0.66) was observed between QDIS-7 and corresponding disease *severity* ratings within all five disease groups. QDIS-7 consistently had higher correlations with other disease-specific measures than with generic physical (*r* = −0.43 to −0.69, median = −0.52) and mental (*r* = −0.38 to −0.51, median = −0.44) summary measures.

**Table 5:** Comparison of relative validity (RV) of QDIS-7 and generic measures in discriminating across severity levels, five disease groups

Disease/Measure	Mean (SD) by Self-Evaluated Severity^a^			F-ratio	RV^b^	95 % CI^c^
	Mild	Moderate	Severe			
Arthritis	(*N* = 688)	(*N* = 564)	(*N* = 214)			
QDIS-7^d^	49.2 (6.88)	57.4 (5.83)	64.9 (5.81)	586.27	1.00	
SF-8 PCS^e^	48.1 (7.60)	40.9 (8.65)	31.2 (8.22)	383.73	0.65	(0.56,0.76)
SF-8 MCS^e^	51.8 (8.08)	48.8 (10.19)	44.7 (11.40)	49.19	0.08	(0.05,0.12)
CKD	(*N* = 189)	(*N* = 56)	(*N* = 33)			
QDIS-7	44.7 (6.96)	53.3 (7.72)	61.3 (8.40)	87.99	1.00	
SF-8 PCS	42.2 (11.10)	37.3 (10.61)	33.3 (7.50)	12.41	0.14	(0.06,0.26)
SF-8 MCS	50.4 (9.43)	45.6 (11.82)	44.4 (11.33)	8.15	0.09	(0.02,0.20)
Cardiovascular	(*n* = 469)	(*n* = 107)	(*n* = 35)			
QDIS-7	48.5 (8.83)	58.9 (6.66)	65.6 (5.60)	123.29	1.00	
SF-8 PCS	41.8 (10.25)	34.2 (8.55)	30.4 (7.21)	43.32	0.35	(0.24,0.50)
SF-8 MCS	49.8 (9.70)	46.5 (10.70)	39.1 (12.74)	21.47	0.17	(0.07,0.30)
Diabetes	(*N* = 870)	(*N* = 317)	(*N* = 58)			
QDIS-7	45.3 (6.42)	51.5 (7.21)	59.0 (5.85)	196.01	1.00	
SF-8 PCS	48.4 (8.76)	44.4 (9.93)	40.6 (11.03)	36.59	0.19	(0.11,0.28)
SF-8 MCS	51.9 (8.08)	48.4 (9.75)	42.6 (11.49)	44.33	0.23	(0.13,0.34)
Respiratory	(*N* = 1106)	(*N* = 297)	(*N* = 109)			
QDIS-7	44.7 (7.10)	56.4 (6.71)	64.3 (7.07)	622.23	1.00	
SF-8 PCS	48.1 (9.46)	41.4 (10.20)	33.4 (10.84)	149.30	0.24	(0.18,0.30)
SF-8 MCS	49.5 (9.34)	46.4 (11.65)	42.2 (12.06)	32.74	0.05	(0.03,0.08)

*Abbreviations: CKD* chronic kidney disease

^a^Severity defined as Mild (None, Mild), Moderate, or Severe (Severe, Very Severe) in response to item *How would you rate the severity of your < condition > in the past 4 weeks?*

^b^Relative validity (RV) is computed as the ratio of the comparator F-statistic over the QDIS-7 F-statistic

^c^Comparator confidence intervals (CI) estimated using bootstrap

^d^QDIS-7 scored so a higher score equals worse health

^e^Norm-based scoring of SF-8 Health Survey summary measures based on a representative probability sample of the US general household population surveyed in 2011, norms (mean = 50, SD = 10) scored so a higher score equals better health

**Table 6:** Correlations of QDIS-7 with disease-specific and generic measures, five disease groups

Disease	N	Disease-specific severity^a^	Disease-specific QOL^b^	Generic Physical^c^	Generic Mental^c^
Arthritis	925	0.72	0.71	−0.69	−0.44
CKD	240	0.66	0.83	−0.44	−0.43
Cardiovascular	542	0.65	0.72, 0.79	−0.52	−0.51
Diabetes	695	0.54	0.72, 0.72	−0.43	−0.49
Respiratory	848	0.74	0.83	−0.58	−0.38

QDIS-7 scored so a higher score equals worse health

*Abbreviations: CKD* chronic kidney disease

^a^Self-rating of disease severity (5 categories, None-Very Severe)

^b^Disease-specific QOL measures are Arthritis: Western Ontario and McMaster Universities Osteoarthritis Index (WOMAC®) Total scale [6]; CKD: Kidney-Disease Quality of Life 36-item instrument (KDQOL-36™) Burden scale [11]; Cardiovascular: Angina/MI-Seattle Angina Questionnaire Quality of Life scale [38] (first entry *N* = 275) and CHF-Minnesota Living with Heart Failure® Questionnaire total scale [39] (second entry, *N* = 267); Diabetes: Problem Areas in Diabetes Scale total scale [41] (first entry) and Diabetes Quality of Life measure total scale [40] (second entry); Respiratory: St. George’s Respiratory Questionnaire total scale [7]. All disease-specific measures are scored so a higher score equals worse health

^c^SF-8 Health Survey physical and mental component summary measures scored so a higher score equals better health

### Responsiveness

In longitudinal analyses, 59.4 % reported the same *pre-ID disease status* at 9-month follow-up and those who changed were more likely better (24.6 %) than worse (16.0 %). Table 7 compares mean changes in QDIS and physical and mental scores across five groups reporting different disease-specific outcomes. In support of the hypothesis that disease-specific QDIS measures are more responsive than generic measures, Table 7 shows a much higher F-ratio (*F* = 29.8, *p* < 0.0001) for QDIS-7 (RV = 1.0) in comparison with generic physical (*F* = 14.2, *p* < 0.0001) and mental (*F* = 2.1, NS) measures, and significantly lower RV estimates (RV = 0.47 and 0.07, respectively). This pattern of results, which supports QDIS responsiveness as a disease-specific measure, was replicated across pre-ID groups analyzed separately with one exception (equivalent QDIS and generic SF-8 PCS RV estimates for OA).

**Table 7:** Responsiveness of QDIS-7 and generic measures in comparisons across groups differing in self-evaluated outcomes during 9-month follow-up, all diseases combined

Measure	Mean change score by self-evaluated outcome^a^					F-ratio	RV^b^	95 % CI^c^
	Much better	Somewhat better	Same	Somewhat worse	Much worse			
	(*N* = 244)	(*N* = 245)	(*N* = 1181)	(*N* = 282)	(*N* = 37)			
QDIS-7^d^	−2.76	−0.04	1.29	3.20	5.87	29.80	1.00	-
SF-8 PCS^d^	1.36	−0.71	−0.28	−3.22	−4.98	14.15	0.47	(0.24,0.85)
SF-8 MCS^d^	1.33	−0.47	0.14	−0.33	−0.91	2.06	0.07	(0.00,0.15)

^a^Self-evaluated change groups were defined as much better, somewhat better, about the same, somewhat worse, or much worse now in response to the question: “Compared to nine months ago, how much better or worse is your < DISEASE > now?”, where DISEASE was the pre-ID condition

^b^Relative validity (RV) is computed as the ratio of the comparator F-statistic over the largest F-statistic for that comparison

^c^Comparator confidence intervals (CI) estimated using bootstrap

^d^Norm-based scoring of all measures based on US general population norms (mean = 50, SD = 10). QDIS-7 scored so a higher score equals worse health; SF-8 scored so a higher score equals better health

### Norms

Norm-based descriptive statistics for five disease-specific severity levels for the combined pre-ID sample are documented in Additional file 7: Figure S4 for use in interpreting cross-sectional results. QDIS means and medians differed substantially and were ordered as hypothesized across severity levels. A noteworthy floor effect was observed only in the least severe (None) group.

## Discussion

QDIS combines the strengths of two traditions within QOL measurement. It harnesses the precision and discriminability of disease-specific assessment with the comprehensiveness of generic QOL assessment. The result is an approach that differs from available disease-specific measures in noteworthy ways. First, and foremost, it standardizes both content and scoring across diseases, which to our knowledge has never been done before. Second, *disease-specific* QOL impact content representation has been increased to be on a par with that of comprehensive *generic* QOL measures. Third, in support of interpreting QDIS as a disease-specific measure, results from this initial evaluation showed that QDIS discriminated across disease severity levels and responded when groups differed in disease-specific outcomes at 9 months markedly better than generic measures. Fourth, QDIS is the first disease-specific measure standardized across diseases and normed in a representative sample of the chronically ill general population.

Standardization began with the content of the same 49 items, differing only in disease-specific attribution. Scoring of a single summary measure was based upon formal tests that confirmed a unidimensional model, consistent with previously-reported results for other disease-specific summary measures [13, 29, 43–45]. Further, the equivalence of parameters across disease groups was sufficient to justify their standardization, and very high (*r* > 0.99) agreement was observed between disease-specific and standardized IRT-based score estimates. Subsequently, an independent test of standardized versus study-specific QDIS-7 item parameters estimated for acute coronary syndrome (ACS) patients showed sufficient IRT invariance to warrant use of standardized parameters in studies comparing QOL impact for ACS and other conditions [17]. In addition to very high (*r* = 0.99) scale-level agreement between ACS-specific and standardized score estimates, this replication is noteworthy because ACS data were collected by telephone interviews versus Internet-based, self-administrations in DICAT.

What is the importance of leveraging broader generic QOL content for purposes of measuring disease-specific impact? By definition, content validity is greater with more complete representation of relevant content areas [3, 8, 68, 69]. Furthermore, representing multiple content areas in QDIS probably results in a more interesting survey administration, in contrast to answering items about the same content multiple times [70]. Respondents also may identify more with one QOL impact description than another. If so, multiple distinct descriptions may be more likely to capture disease impact and expand the usefulness of information available for interpreting research results and for clinicians and patients to discuss.

Like *all* measures relying on disease-specific attributions, QDIS assumes that respondents with multiple chronic conditions (MCC) can validly differentiate the specific impact of one disease from that of others, a rarely tested assumption. Although our initial results comparing measures differing in attributions to a specific disease versus health in general support this assumption, current study methods did not test it directly. For example, the greater validity and responsiveness observed for QDIS over generic measures could reflect the impact of a comorbid condition. This crucial issue has been addressed in a parallel study [71] of adults with MCC. Results from multitrait-multimethod (MTMM) tests of construct validity [72] based on analysis of DICAT data for 4480 respondents with MCC strongly support the assumption that adults can validly differentiate the specific impact of one condition from that of others. Briefly, results from MTMM tests of up to 26 comorbid conditions within each of eight pre-ID conditions demonstrated convergent validity; correlations among three methods (QDIS, severity, symptoms) of measuring the *same condition* were substantial (*r* = 0.38 to 0.84, median = 0.53) across pre-ID conditions. In contrast, as hypothesized for discriminating measures, correlations between methods of measuring *different conditions* were significantly lower than corresponding convergent correlations in 833 of 924 (90.2 %) tests; exceptions were most often observed for comorbid conditions in the same clinical area. It follows from these results and those reported in this paper that the standardized QDIS approach based on attributions to specific diseases warrants further tests of its use in quantifying and comparing each disease as well as aggregating scores to estimate the cumulative burden of multiple diseases, thereby addressing an important measurement gap [69].

Historically, an advantage of generic QOL measures over disease-specific measures has been the availability of general population norms for use in interpreting generic outcomes. However, this tradeoff is unnecessary to the extent that disease-specific populations can be defined and sampled. A practical limitation to widespread standardization and norming has been the many different disease-specific measures. By standardizing both content and scoring, QDIS enables a practical approach to the norm-based interpretation of disease-specific QOL impact throughout the chronically ill population. To make norm-based interpretation easier, scores were transformed to have a mean of 50 and SD of 10 in the US chronically ill household population using a T-score transformation, such as that adopted for the SF-36 [73], SF-12® [74] and SF-8 [23] Health Surveys and PROMIS® [75]. By placing all disease-specific scores on the same QOL impact metric, clinicians can better understand the implications of differences in disease severity with a level of specificity that is not possible with a generic measure and researchers can aggregate patient scores for predictive and outcome analyses across diseases.

### Modeling issues

Considerable art is involved in the application of measurement theory and methods to the measurement of QOL impact. Accordingly, different interpretation of the multiple criteria applied here or the application of different methods might have led to different selections of items for the 7-item static form (QDIS-7) and the 25-item bank. In our Methods and Results sections, we have attempted to explain the logic that was applied in considering item-specific evidence of many types. To facilitate other choices and replications in other disease groups, we have documented results for the entire 49-item bank.

The strong support for a 1-factor model observed in every disease group studied is consistent with previous findings for QOL items making attributions to specific conditions [13, 29, 43]. It is also in sharp contrast to measurement models for generic items and scales that confirm conceptually- and empirically-distinct subdomains and higher-order physical and mental factors [73, 76]. In marked contrast, it appears that adults asked to focus on a specific condition make QOL attributions more on the basis of differences in the overall severity and QOL impact of each condition and less on the basis of the different aspects of QOL (e.g., physical, emotional, role/social).

QDIS scored using the classical method of summated ratings [77] and IRT item parameter estimates correlated very highly throughout the score range in every disease group. Hence, scores estimated using both methods can be transformed (mean = 50, SD = 10) throughout the chronically ill US population. However, well-documented and noteworthy advantages of IRT models, such as more accurate CAT-based estimates of *individual* scores at every level of theta, would be lost in the absence of IRT-based item parameters.

### Alternate forms

Because electronic data capture is not always possible, the QDIS-7 fixed-length form was evaluated in parallel with CAT as in previous studies [57, 62, 65]. In support of the direct comparability of norm-based scoring of both approaches, the correlation between the static QDIS-7 and QDIS-CAT-6 scores ranged from 0.97 to 0.98 across the five disease groups studied and mean estimates were nearly identical across measures. We also extended the adaptive logic at the 9-month follow-up by using CAT only for those patients suspected of scoring in the most impaired range and relying on a noisier but unbiased single-item QDIS estimate (QL1) for those showing lower or no impact. The gains in efficiency included a nearly 80 % reduction in respondent burden while still achieving the hypothesized superiority in responsiveness over generic outcome measures. This alternative to static short-form and routine fixed-length CAT measurement warrants further study, particularly for purposes of individual-level applications requiring greater reliability than group-level comparisons.

The single-item QDIS estimate (QL1) correlated 0.89 with the 49-item bank theta score and produces unbiased (although coarse) estimates of disease-group means across population surveys and large-group studies if scored using the recommended T-score transformation. This single-item measure has also been shown to achieve convergent and discriminant validity among adults with multiple chronic conditions (MCC). Therefore, it may provide a practical pathway to a standard global QOL impact measure that uses disease-specific attribution to measure total MCC impact. Such an aggregate measure may be a valid addition to the toolkit for adjusting for differences in case mix in observational studies of patient-reported outcomes.

### Limitations

Although replications across diseases are rare for new approaches to QOL measurement, it is a limitation that only nine conditions in five disease groups were the basis of initial QDIS development and validation. The consistency of results across diseases suggests that the findings are likely to generalize to other physical conditions. Analyses of subgroups—OA and RA within arthritis, asthma and COPD within respiratory, and angina, MI and CHF within cardiovascular—replicated findings reported here, although with some limitations due to smaller samples. However, all of the pre-ID disease groups were physical health conditions, and the omission of any pre-ID mental health groups is a noteworthy limitation of the current study. Self-reported depression, not analyzed here, was prevalent (13–26 %) across the pre-ID groups and was shown elsewhere to be validly measured by QDIS as a comorbid condition [71]. We recommend extension of future studies to include mental and other physical diseases.

Another potential limitation is reliance entirely on self-reports for disease severity and legacy disease-specific measures. Although self-report methods have been useful in validation [16, 75] results may have been different if “criteria” had been based on independent clinical evaluations. Although any self-report bias is unlikely to account for the superior discriminant validity observed for QDIS over generic measures, QDIS should be evaluated in relation to more objective clinical criteria, which are also likely to make interpretation guidelines more useful clinically. Examples of more independent clinical validation include a recent trial using QDIS with attribution to smoking; QDIS correlated substantially higher with four biomarkers of smoking exposure [78] and discriminated between current and former smokers much better [79] than generic SF-36v2® Health Survey measures. Similarly, QDIS with attribution to kidney disease discriminated across clinically-defined stages of CKD better than the SF-12 [65].

The lack of new patient involvement in selecting and modifying QDIS items is a noteworthy potential limitation of the current study. Although QDIS item selection and modification benefited from previous qualitative studies of phrases in the QDIS items, it is possible that de novo qualitative studies would lead to further improvements, if changes in attributions from health to a specific condition materially change item meaning or clarity. To what extent does changing the attribution of QOL impact from health in general to a specific disease require additional patient involvement in qualitative evaluation [80]? The advantage of more such evidence is the opportunity to add findings from qualitative studies to the rich array of evidence in Table 2 and to better understand the inevitable tradeoffs in choosing among items. However, single-disease studies often ignore the presence of multiple chronic conditions. Accordingly, we recommend that qualitative research be conducted systematically among patients with MCC for QDIS and other measures that use disease-specific attributions.

### Recommendations for future research

In addition to the issues noted above, the psychometric properties of items selected for administration in static QDIS short forms or by CAT warrant further study. Our observation that some of the poorest fitting items in terms of traditional fit statistics were the most predictive and most utilized by CAT led us to consider multiple criteria in selecting items for QDIS-7 and the 25-item bank, including information functions at the most prevalent score levels. Further, more in-depth tests of item- and scale-level parameter invariance analogous to the ACS study [17] are likely to be informative.

To the extent that current study findings are generalizable to other conditions for which comprehensive disease-specific QOL measures are not readily available, QDIS may enable a substantial short-cut to achieving disease-specific QOL impact estimates. Examples include applications to rare diseases and the evaluations of orphan drugs. Patient and clinician input in establishing clear and familiar terminology for disease-specific attribution would be a prerequisite and assumptions underlying scoring, reliability and validity would need to be evaluated, as always should be done for a new disease application of a standardized measure. However, for conditions lacking a summary QOL impact measure, QDIS may be a practical solution that does not require years of development and considerable resources.

## Conclusions

Overall, this pursuit of disease-specific QOL measurement innovation appears to be on the right track toward filling the conceptual and methodological gaps between disease-specific symptoms that do not measure quality of life and generic QOL measures that do not measure disease-specific outcomes. By integrating the richness of generic QOL item content with disease-specific attributions and by standardizing scoring metrics, QDIS achieves some of the advantages of both disease-specific and generic PRO measurement traditions. The result is a new method for comparing outcomes across diseases while retaining the advantages of disease-specific measures. The broader representation of item content in QDIS may expand knowledge about the various ways in which specific diseases impact patients’ quality of life and the benefits of their treatment. To facilitate its use, information about QDIS and permission to use it for scholarly and commercial applications is available at www.jwrginc.com.

## Abbreviations

ACS, acute coronary syndrome; CAT, computerized adaptive testing; CFA, confirmatory factor analysis; CFI, coefficient of fit index; CKD, chronic kidney disease; DICAT, Computerized Adaptive Assessment of Disease Impact project; DIF, differential item functioning; GPCM, generalized partial credit model; ICC, intraclass correlation coefficient; IRT, item response theory; MCC, multiple chronic conditions; MCS, mental component summary; MGCFA, multi-group confirmatory factor analysis; MOS, Medical Outcomes Study; OLR, ordinal logistic regression; PCS, physical component summary; PRO, patient-reported outcome; PROMIS, Patient Reported Outcomes Measurement Information System; QDIS, Quality of life Disease Impact Scale; QDIS-7, 7-item short-form of QDIS; QDIS-CAT-6, 6-item adaptive short form of QDIS; QOL, quality of life; RMSEA, root mean square error of approximation; RV, relative validity; SF-12, SF-12 Health Survey; SF-36, SF-36 Health Survey; SF-8, SF-8 Health Survey; WLSMV, weighted least squares estimator with mean and variance adjustment.

## Additional files

Additional file 1: Table S1. Comparison of relative validity (RV) of QDIS (7-item Static and 6-item CAT) and generic measures in discriminating across severity levels, five disease groups. (PDF 104 kb) Additional file 2: Table S2. Correlations of QDIS-7 (7-item Static and 6-item CAT) with disease-specific and generic measures, five disease groups. (PDF 155 kb) Additional file 3: Figure S1. Plots of QDIS disease-specific and standardized slopes. (PDF 123 kb) Additional file 4: Figure S2. Plots of QDIS disease-specific and standardized thresholds. (PDF 122 kb) Additional file 5: Table S3. Correlations among disease-specific and standardized IRT parameters^a^. (PDF 90.5 kb) Additional file 6: Figure S3. Plots of QDIS theta scores estimated using disease-specific and standardized parameters. (PDF 196 kb) Additional file 7: Figure S4. Plot of QDIS-7 medians and ranges for standardized scores and percentile ranks by severity level, all disease groups combined. (PDF 130 kb)
