# Supplementary material for: Standardizing disease-specific quality of life measures across multiple chronic conditions: development and initial evaluation of the QOL Disease Impact Scale (QDIS®)
Source: Health Qual Life Outcomes. 2016 Jun 2;14:84. doi: 10.1186/s12955-016-0483-x (PMC4890258; doi:10.1186/s12955-016-0483-x)
Supplement: Additional file 1: Table S1. — Comparison of relative validity (RV) of QDIS (7-item Static and 6-item CAT) and generic measures in discriminating across severity levels, five disease groups. (PDF 104 kb) [file 12955_2016_483_MOESM1_ESM.pdf]

**Additional File 1: Table S1** Comparison of Relative Validity (RV) of QDIS (7-item Static and 6-item CAT) and Generic Measures in Discriminating Across Severity Levels, Five Disease Groups

| Disease/ Measure | Mean (SD) by Self-Evaluated Severity[a] | | | F-ratio | RV[b] | 95% CI[c] |
|---|---|---|---|---|---|---|
| | Mild | Moderate | Severe | | | |
| Arthritis | (N=499) | (N=406) | (N=158) | | | |
| QDIS-7[d] | 48.8 (7.04) | 57.0 (5.99) | 65.1 (5.85) | 434.04 | 1.00 | |
| CAT-6[d] | 49.4 (7.17) | 57.1 (5.64) | 64.4 (5.70) | 379.44 | 0.87 | (.83, .93) |
| SF-8 PCS[e] | 47.9 (7.73) | 41.2 (8.59) | 31.3 (8.39) | 262.63 | 0.61 | (.50, .72) |
| SF-8 MCS[e] | 51.3 (8.58) | 48.6 (10.02) | 43.7 (11.47) | 38.50 | 0.09 | (.05, .14) |
| CKD | (N=173) | (N=46) | (N=30) | | | |
| QDIS-7 | 44.3 (6.91) | 52.0 (7.26) | 61.2 (8.72) | 79.21 | 1.00 | |
| CAT-6 | 44.8 (7.02) | 52.0 (7.07) | 61.0 (7.91) | 74.39 | 0.94 | (.84,1.07) |
| SF-8 PCS | 42.3 (11.17) | 37.1 (10.66) | 32.9 (7.18) | 12.20 | 0.15 | (.07, .31) |
| SF-8 MCS | 50.3 (9.19) | 46.3 (11.29) | 44.3 (11.80) | 6.71 | 0.08 | (.02, .19) |
| Cardiovascular | (n = 431) | (n = 97) | (n = 32) | | | |
| QDIS-7 | 48.4 (8.79) | 58.9 (6.63) | 65.3 (5.40) | 113.33 | 1.00 | |
| CAT-6 | 48.8 (9.03) | 58.8 (6.72) | 65.3 (5.20) | 100.47 | 0.89 | (.82, .94) |
| SF-8 PCS | 41.8 (10.25) | 34.1 (8.57) | 30.0 (7.34) | 40.59 | 0.36 | (.23, .52) |
| SF-8 MCS | 49.9 (9.55) | 46.5 (10.41) | 39.7 (11.51) | 18.83 | 0.17 | (.07, .30) |
| Diabetes | (N=569) | (N=212) | (N=45) | | | |
| QDIS-7 | 44.4 (6.42) | 51.2 (7.63) | 58.9 (5.95) | 152.73 | 1.00 | |
| CAT-6 | 44.5 (6.23) | 51.0 (7.52) | 58.0 (6.43) | 142.47 | 0.93 | (.86, 1.01) |
| SF-8 PCS | 48.3 (8.91) | 44.1 (9.97) | 40.9 (11.41) | 25.25 | 0.17 | (.08, .27) |
| SF-8 MCS | 51.5 (8.32) | 47.7 (10.18) | 42.3 (12.00) | 31.08 | 0.20 | (.11, .33) |
| Respiratory | (N=809) | (N=230) | (N=83) | | | |
| QDIS-7 | 44.1 (7.07) | 56.2 (6.67) | 64.9 (7.26) | 524.31 | 1.00 | |
| CAT-6 | 44.5 (7.08) | 56.4 (6.60) | 64.1 (6.86) | 488.29 | 0.93 | (.89, .98) |
| SF-8 PCS | 47.9 (9.57) | 41.3 (10.34) | 32.8 (10.29) | 114.33 | 0.22 | (.16, .28) |
| SF-8 MCS | 49.2 (9.56) | 46.2 (11.76) | 40.8 (11.80) | 29.14 | 0.06 | (.03, .09) |

*Abbreviations: CKD* chronic kidney disease

[a] Severity defined as Mild (None, Mild), Moderate, or Severe (Severe, Very Severe) in response to item *How would you rate the severity of your <condition> in the past 4 weeks?*

[b] Relative validity (RV) is computed as the ratio of the comparator F-statistic over the QDIS-7 F-statistic.

[c] Comparator confidence intervals (CI) estimated using bootstrap.

[d] QDIS-7 and CAT-6 scored so a higher score equals worse health.

[e] Norm-based scoring of SF-8 Health Survey summary measures based on a representative probability sample of the US general household population surveyed in 2011, scored so a higher score equals better health.

Source: Ware JE, Gandek B, Guyer R, Deng N. Standardizing Disease-specific Quality of Life Measures Across Multiple Chronic Conditions: Development and Initial Evaluation of the QOL Disease Impact Scale (QDIS®). *Health and Quality of Life Outcomes*, 2016.
