# Supplementary material for: Standardizing disease-specific quality of life measures across multiple chronic conditions: development and initial evaluation of the QOL Disease Impact Scale (QDIS®)
Source: Health Qual Life Outcomes. 2016 Jun 2;14:84. doi: 10.1186/s12955-016-0483-x (PMC4890258; doi:10.1186/s12955-016-0483-x)
Supplement: Additional file 2: Table S2. — Correlations of QDIS-7 (7-item Static and 6-item CAT) with disease-specific and generic measures, five disease groups. (PDF 155 kb) [file 12955_2016_483_MOESM2_ESM.pdf]

Additional File 2: Table S2 Correlations of QDIS-7 (7-item Static and 6-item CAT) with Disease-Specific and Generic Measures, Five Disease Groups

Disease Group	N	Disease-specific severity[a]	Disease-specific QOL[b]	Generic Physical[c]	Generic Mental[c]
Arthritis					
QDIS-7	925	0.72	0.71	-0.69	-0.44
CAT-6	925	0.70	0.70	-0.68	-0.43
CKD					
QDIS-7	240	0.66	0.83	-0.44	-0.43
CAT-6	240	0.65	0.82	-0.44	-0.43
Cardiovascular					
QDIS-7	542	0.65	0.72, 0.79	-0.52	-0.51
CAT-6	542	0.63	0.72, 0.78	-0.52	-0.50
Diabetes					
QDIS-7	695	0.54	0.72, 0.72	-0.43	-0.49
CAT-6	695	0.53	0.70, 0.71	-0.43	-0.49
Respiratory					
QDIS-7	848	0.74	0.83	-0.58	-0.38
CAT-6	848	0.73	0.83	-0.59	-0.38

Correlations between QDIS-7 and CAT-6 measures were arthritis (0.97), CKD (0.97), cardiovascular (0.98), diabetes (0.97), and respiratory (0.98). QDIS-7 scored so a higher score equals worse health.

Abbreviations: CKD chronic kidney disease

[a] Self-rating of disease severity (5 categories, None-Very Severe).

[b] Disease-specific QOL measures are Arthritis: Western Ontario and McMaster Universities Osteoarthritis Index (WOMAC®) Total scale [6]; CKD: Kidney-Disease Quality of Life 36-item instrument (KDQOL-36™) Burden scale [11]; Cardiovascular: Angina/MI-Seattle Angina Questionnaire Quality of Life scale [38] (first entry n=275) and CHF-Minnesota Living with Heart Failure® Questionnaire total scale [39] (second entry, n=267); Diabetes: Problem Areas in Diabetes Scale total scale [41] (first entry) and Diabetes Quality of Life measure total scale [40] (second entry); Respiratory: St. George's Respiratory Questionnaire total scale [7]. All disease-specific measures are scored so a higher score equals worse health.

[c] SF-8 Health Survey physical and mental component summary measures scored so a higher score equals better health.

Source: Ware JE, Gandek B, Guyer R, Deng N. Standardizing Disease-specific Quality of Life Measures Across Multiple Chronic Conditions: Development and Initial Evaluation of the QOL Disease Impact Scale (QDIS®). *Health and Quality of Life Outcomes*, 2016.
