# Supplementary material for: Standardizing disease-specific quality of life measures across multiple chronic conditions: development and initial evaluation of the QOL Disease Impact Scale (QDIS®)
Source: Health Qual Life Outcomes. 2016 Jun 2;14:84. doi: 10.1186/s12955-016-0483-x (PMC4890258; doi:10.1186/s12955-016-0483-x)
Supplement: Additional file 5: Table S3. — Correlations among disease-specific and standardized IRT parametersa. (PDF 90.5 kb) [file 12955_2016_483_MOESM5_ESM.pdf]

Additional File 5: Table S3 Correlations Among Disease-Specific and Standardized IRT Parameters[a]

Disease	Slopes					Thresholds				
	Arthritis	CKD	Cardio-vascular	Dia-betes	Respi-ratory	Arthritis	CKD	Cardio-vascular	Dia-betes	Respi-ratory
CKD	0.786					0.904				
Cardiovascular	0.834	0.811				0.968	0.913			
Diabetes	0.801	0.841	0.826			0.918	0.916	0.923		
Respiratory	0.878	0.793	0.892	0.803		0.983	0.913	0.978	0.918	
Standardized	0.934	0.877	0.928	0.897	0.957	0.993	0.930	0.984	0.942	0.992

Abbreviations: CKD chronic kidney disease

Sample size: Arthritis N=1,574; CKD N=299; Cardiovascular N=639; Diabetes N=1,326; Respiratory N=1,580; Standardized (total combined across diseases) N=5,418.

[a] All entries are product-moment correlations. Correlations between disease-specific and standardized parameters are not independent.

Source: Ware JE, Gandek B, Guyer R, Deng N. Standardizing Disease-specific Quality of Life Measures Across Multiple Chronic Conditions: Development and Initial Evaluation of the QOL Disease Impact Scale (QDIS®). *Health and Quality of Life Outcomes*, 2016.
