# Supplementary material for: Standardizing disease-specific quality of life measures across multiple chronic conditions: development and initial evaluation of the QOL Disease Impact Scale (QDIS®)
Source: Health Qual Life Outcomes. 2016 Jun 2;14:84. doi: 10.1186/s12955-016-0483-x (PMC4890258; doi:10.1186/s12955-016-0483-x)
Supplement: Additional file 6: Figure S3. — Plots of QDIS theta scores estimated using disease-specific and standardized parameters. (PDF 196 kb) [file 12955_2016_483_MOESM6_ESM.pdf]

Additional File 6: Figure S3 Plots of QDIS Theta Scores Estimated Using Disease-specific and Standardized Parameters

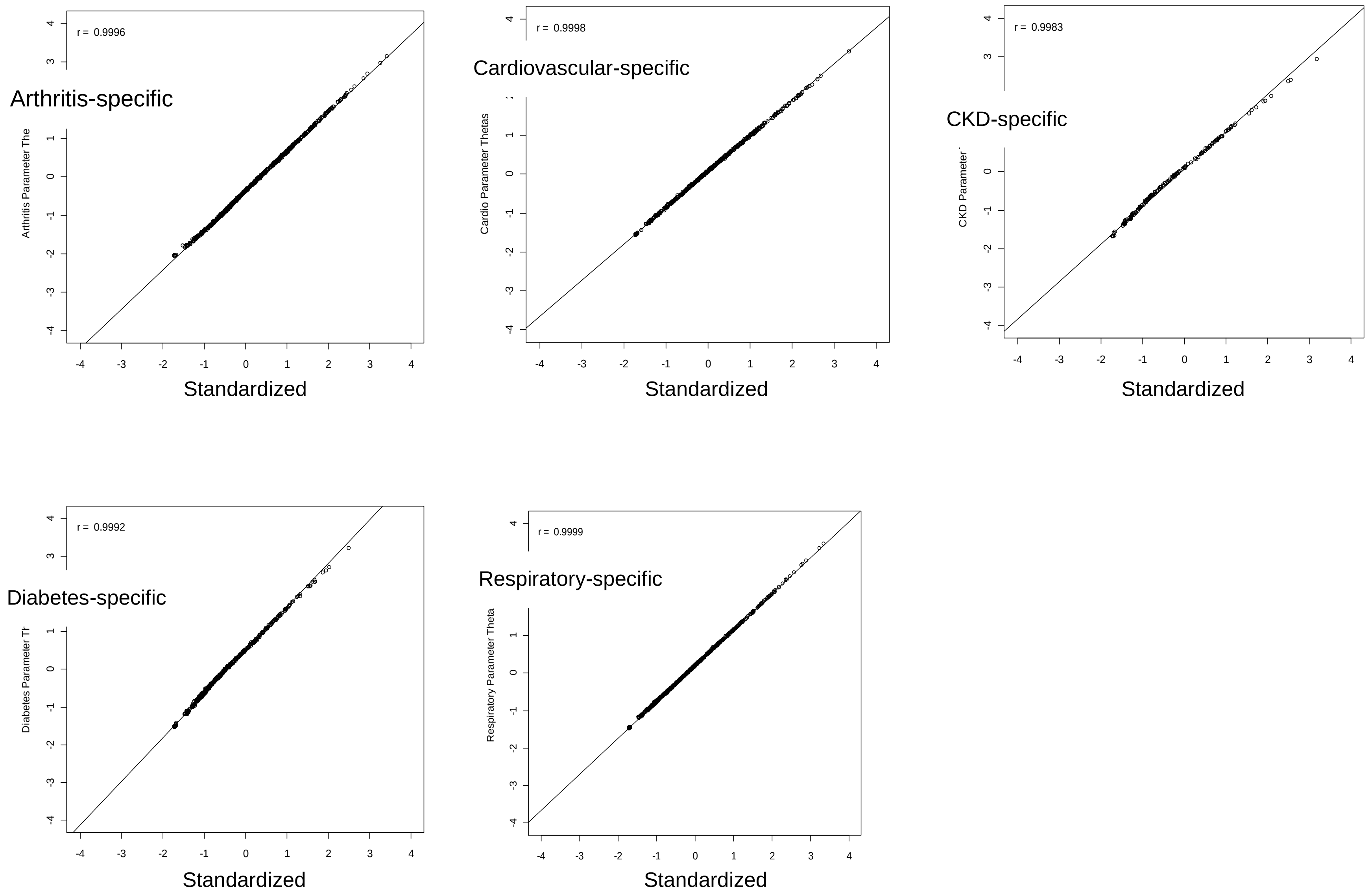

Source: Ware JE, Gandek B, Guyer R, Deng N. Standardizing Disease-specific Quality of Life Measures Across Multiple Chronic Conditions: Development and Initial Evaluation of the QOL Disease Impact Scale (QDIS®). *Health and Quality of Life Outcomes*, 2016
