# Supplementary material for: Standardizing disease-specific quality of life measures across multiple chronic conditions: development and initial evaluation of the QOL Disease Impact Scale (QDIS®)
Source: Health Qual Life Outcomes. 2016 Jun 2;14:84. doi: 10.1186/s12955-016-0483-x (PMC4890258; doi:10.1186/s12955-016-0483-x)
Supplement: Additional file 7: Figure S4. — Plot of QDIS-7 medians and ranges for standardized scores and percentile ranks by severity level, all disease groups combined. (PDF 130 kb) [file 12955_2016_483_MOESM7_ESM.pdf]

Additional File 7: Figure S4 Plot of QDIS-7 Medians and Ranges for Standardized Scores and Percentile Ranks by Severity Level, All Disease Groups Combined

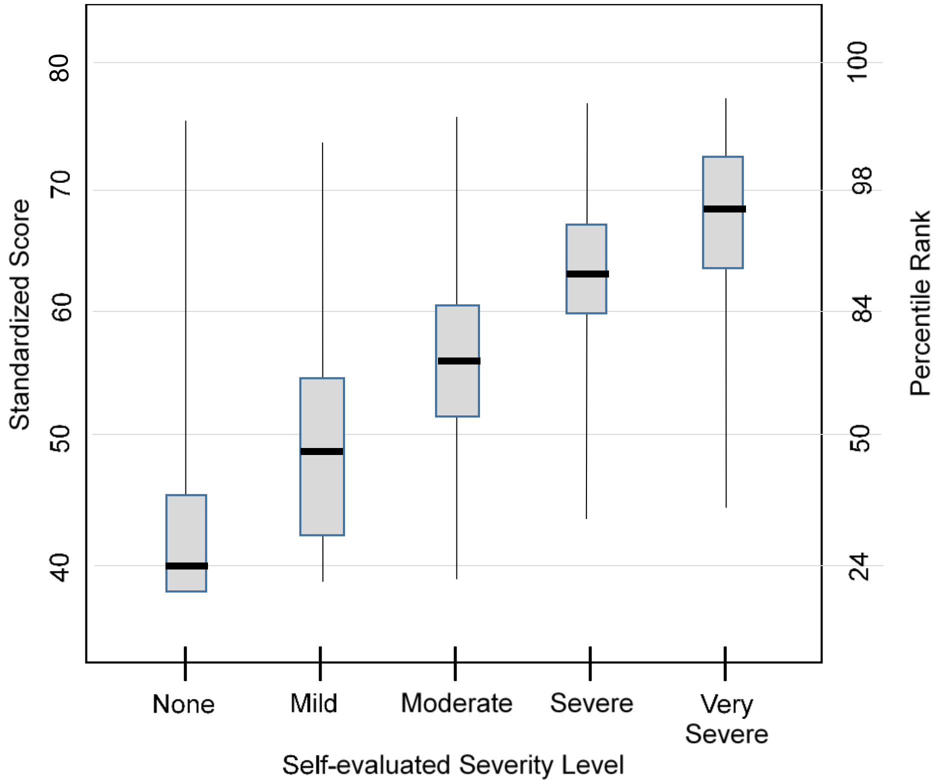

Note: Bold lines are medians; vertical lines are observed ranges; gray boxes are interquartile ranges.

	Severity Level				
	**None**	**Mild**	**Moderate**	**Severe**	**Very Severe**
N	1,205	2,271	1,396	382	97
Mean	42.43	48.38	55.79	62.68	67.86
Standard deviation	5.97	7.21	7.02	6.06	7.21
25th percentile	38.08	42.06	51.83	59.67	63.28
50th percentile	39.76	48.85	56.25	63.15	68.48
75th percentile	45.62	53.70	60.80	66.63	73.10
Range	38.1-77.3	38.1-74.0	38.1-76.0	43.0-77.3	42.9-77.3
% ceiling (worst)	<1	0	0	<1	16.5
% floor (best)	45.9	16.3	14	<1	<1

Source: Ware JE, Gandek B, Guyer R, Deng N. Standardizing Disease-specific Quality of Life Measures Across Multiple Chronic Conditions: Development and Initial Evaluation of the QOL Disease Impact Scale (QDIS®). *Health and Quality of Life Outcomes*, 2016.
